# Evaluating Penetration Ability of *Plodia interpunctella* (Hübner) (Lepidoptera: Pyralidae) Larvae into Multilayer Polypropylene Packages

**DOI:** 10.3390/insects9020042

**Published:** 2018-04-18

**Authors:** Deanna S. Scheff, Blossom Sehgal, Bhadriraju Subramanyam

**Affiliations:** 1 Department of Grain Science and Industry, Kansas State University, Manhattan, KS 66506, USA; sbhadrir@k-state.edu; 2 United States Department of Agriculture-Agricultural Research Service, Center for Grain and Animal Health Research, Manhattan, KS 66502, USA

**Keywords:** *Plodia interpunctella*, stored-product insects, package penetration, package resistance, energy bar damage

## Abstract

The larvae of the Indian meal moth, *Plodia interpunctella* (Hübner), can invade or penetrate packaging materials and infest food products. Energy bars with three polypropylene packaging types were challenged with eggs (first instars), third instars, and fifth instars of *P. interpunctella* to determine package resistance at 28 °C and 65% r.h. The packing types were also challenged with two male and two female pupae of *P. interpunctella* under similar conditions in order to determine which package provided the greatest protection against larval penetration. Samples infested with eggs, third instars, and pupae were evaluated after 21 days and 42 days to count the number of larvae, pupae, and adults found inside the packages. Packages challenged with fifth instars were observed after 21 days to count the number of larvae, pupae, and adults inside each package. The number and diameter of the holes were determined in each package, followed by the amount of damage sustained to the energy bar. Third and fifth instars showed a higher tendency to penetrate all of the packaging types. First instars showed a reduction in package penetration ability compared with third and fifth instars. The increase in exposure time resulted in an increase in the damage sustained to the energy bars. Among packaging types, the thickest package (Test A) was most resilient to penetration by all of the larval stages. In conclusion, energy bar manufacturers need to invest more effort into improving packaging designs, creating thicker gauge films, or advancing odor barrier technology, in order to prevent penetration and infestation by *P. interpunctella* larvae.

## 1. Introduction

The presence of stored-product insects in ready-to-eat packaged products causes product losses, decreases in consumer confidence, and the possibility of allergic reactions in sensitive individuals [1]. In addition, economic losses faced by the food industry include fines, penalties by government agencies, the possibility of litigation by consumers who may suffer from eating infested products, and insurance claims [2]. The infestation of packaged products occurs due to insect contamination in raw ingredients during the manufacturing process, storage in warehouses, transportation, and storage in retail environments and consumer homes [3]. Stored-product insects are a persistent problem in retail stores, warehouses, and grain-processing facilities [4]. The infestation of packaged products at the retail level arises from stores receiving infested product from the manufacturer, insects entering the retail store through open doors/windows, or insect populations that are established within the store finding the products [5]. In the retail marketplace, insects will contaminate and cause damage to a variety of food products such as candy bars, flour, granola, and pet food [4]. Food processors may take all possible precautions to prevent infestations in their packaged products during manufacturing, but they have little or no control over their product during shipping and storage in warehouses and retail environments [5,6]. Retail stores need to employ a multi-barrier approach to pest management in order to reduce or eliminate infestations. Stock rotation, food-baited and pheromone-baited insect traps, the inspection of incoming products, and good sanitation practices are some examples of an effective pest management program [1,4].

Traditionally, retailers and pest management professionals relied on chemical pesticides to deal with infestation issues in retail environments [4]. In recent years, there has been a trend in the industry to find alternative measures to deal with infestation problems. Manufactures have begun using alternative methods to protect their packaged products such as odor barriers, insect growth regulators (IGR), and insect-resistant packaging [7]. In order to create insect-resistant packaging, one needs to understand the mechanisms by which insects penetrate product packaging. A thorough understanding of where, when, and how infestations occur in the packaged product will help in identifying critical aspects of the packaging material that need to be addressed [8]. Currently, a limited amount of data are available that scientifically document the insect penetration of commercially packaged food products, especially by the Indian meal moth, *Plodia interpunctella* (Hübner), a common insect pest in retail environments [5].

The stored-product insects that affect packaged products are cosmopolitan [6]. Two scenarios have been proposed that explain the presence of stored-product insects in packaged foods: (1) insects are present in the product before packaging, or (2) insects invade or penetrate the product after packaging [8]. Based on these two scenarios, stored-product insects are classified as package invaders and package penetrators [6]. Invaders enter a package through a pre-existing opening such as a tear, seam failure, or puncture [9]. Approximately 75% of infestations occur due to insects invading a package [10]. Invaders have weakly developed mouthparts in the larval and adult stages [11]. Newly hatched larvae are of most concern because they possess the ability to invade holes as small as 0.1 mm in diameter [11]. Penetrating insects can bore through flexible packaging materials due to their large and powerful mouthparts [9,11]. The ability of insects to penetrate packaging materials depends on the species, life stage, material construction, and the presence of creases, scratches, or tears on the package [12,13]. Insect species that are penetrators are most dangerous in the larval stage [11].

*P. interpunctella* can attack a wide range of stored products [14]. *P. interpunctella* has historically been among the world’s most economically important stored-product insect pest of raw and processed foods, and is associated with food and feed processing plants, warehouses, and retail environments [2,4,5]. *P. interpunctella* larvae are associated with 179 different food commodities in 48 different countries spanning six continents [15]. In a survey of four Hawaiian Islands, 54 species of beetles and 11 species of moths were identified, of which *P. interpunctella* was the most frequently observed insect [2]. *P. interpunctella* was the most common and abundant insect found in five retail stores in north–central Florida and in eight grocery stores in Oklahoma [4,16].

Adults of *P. interpunctella* are capable of laying 100–300 eggs per female [17]. Newly-hatched larvae wander in search of food. While larvae are mobile, they leave behind a sticky silk, and in this webbing, the fecal material and cast skins of earlier instars may become entrapped [17]. Newly hatched larvae are able to invade films with pinholes of 0.293 mm or larger, but cannot invade pinholes of 0.173 mm or smaller, and are unable to penetrate polyethylene films of 20 µm thickness [18]. Second and fifth instars of *P. interpunctella* have shown the ability to chew through Kraft paper (114 µm thick), polyethylene (25.4 µm), and aluminum foil (16.5 µm) when held without food [19]. Fifth instars of *P. interpunctella* were unable to penetrate polypropylene (28 µm) pouches, but they were able to penetrate 63% of polyvinyl chloride (25 µm) pouches. In addition, 25% of polyvinyl chloride (25 µm) pouches were penetrated by first instars, indicating that older instars are stronger penetrators compared with first instars [20]. In the first study, second and fifth instars were unable to penetrate 25.4 µm polyvinyl chloride pouches when food was provided; however, when food was not present, fifth instars penetrated 33% of the pouches [19]. The absence of food induced penetration due to larvae searching for food. Both studies demonstrated that older instars show a greater tendency to penetrate packages than younger instars, especially in the absence of food. Limited data are available on the penetration ability of various larval stages of *P. interpunctella* in multilayer polypropylene packaging. Understanding the ability of several larval instars to penetrate packaging material will aid food manufactures in selecting appropriate packaging materials and thus protecting their products. The previous studies on *P. interpunctella* penetration/invasion were conducted on single-layer polymer films. Multilayer films consisting of two or more liquid polymers coextruded to form a singular film containing the properties of each polymer. Multilayer films can also consist of two or more polymers held together with an adhesive. Multilayer films are designed to maximize the strengths of the polymer constituents, and are thought to increase packaging durability.

There is no standard methodology for evaluating packaging integrity against *P. interpunctella* penetration, regardless of the polymer matrix. Therefore, the objective of this research was to evaluate the susceptibility of three types of commercial polypropylene-packaged energy bars to penetration by high population densities of *P. interpunctella* first, third, and fifth instars, when packages were challenged without food. Secondly, this experiment is designed to provide criterion, guidelines, and methodology to use when testing packaging integrity against *P. interpunctella* in future research efforts on insect-resistant packaging.

## 2. Materials and Methods

### 2.1. Insects

Cultures of *P. interpunctella*, which have been in rearing since 1999 in the Department of Grain Science and Industry at Kansas State University, Manhattan, Kansas, United States of America (USA), were used in this experiment. Insects were reared on a diet consisting of 1000 g of poultry mash, 150 mL of glycerol, 150 mL of honey, and 75 mL of distilled water at 28 °C, 65% r.h., and 14:10 L:D photoperiod [21].

### 2.2. Properties of Packaging Films

A commercial energy bar manufacturer provided three packaging film types, each containing a single energy bar, which were used in this study. All of the packaging samples were visually the same in appearance, and contained the same type of energy bar. All of the packages were comprised of multilayer films. Packaging films included Test A, which was made of 15.24 µm oriented polypropylene/30.48 µm metalized cast polypropylene (total thickness, 48.7 µm); Test B, which was made of 15.24 µm oriented polypropylene/27.90 µm metalized oriented polypropylene (total thickness, 43.14 µm), and Test C, which was made of 15.24 µm oriented polypropylene/25.40 µm metalized cast polypropylene (total thickness, 40.64 µm). All of the other packaging attributes were considered proprietary information, and cannot be disclosed here.

All of the packages were evaluated visually for pre-existing rips, tears, or holes in the packaging material before use in all of the experiments, thus eliminating *P. interpunctella* larvae from invading the package due to a pre-existing entry point. Seam integrity was examined to determine if a hole, leak, or imperfect seal was present. Further seam integrity testing was not warranted, because such testing would compromise packaging material prior to use.

### 2.3. Experiment 1: Effect of Larval Density on the Penetration Ability of First Instar P. interpunctella 

Male and female moths were collected from cultures and introduced into a 0.95-L glass jar fitted with a mesh screen and filter paper. The glass jar was inverted over a 9-cm glass Petri dish, and moths were allowed to mate and oviposit. Eggs (0–24 h old) were collected and counted under a stereomicroscope, and densities of 50, 200, or 400 eggs were added to a 0.45-L glass jar containing a single package type—Test A, B, or C—which was placed vertically inside the glass jar and closed with a metal lid fitted with a mesh screen and filter paper. Insects were held without food to compel larvae to penetrate packages in order to obtain access to a food source. Previous research has shown that insects held without food had a higher tendency to penetrate packages compared with insects held with a food source [19,22]. This experimental arrangement was used to simulate a low, medium, and high density population that may be present in a retail store.

Eggs were used to represent first instars, in order to prevent handling losses. Jars were held at 28 °C and 65% r.h. Observations were made after 21 days and 42 days post-infestation. Each egg density, package type, and exposure duration was replicated five times. The average number of eggs that hatched was determined as follows: three replicates of 100 eggs each of *P. interpunctella* were collected and placed in glass Petri dishes. Dishes were placed at 28 °C and 65% r.h. and examined after 7 days [23]. The percentage of eggs that hatched out of the total (100) was calculated. The mean ± SE egg hatch was 79.3 ± 2.4%.

After 21 days and 42 days post-infestation, packages were removed from jars and examined for the number of larvae, pupae, and adults present inside each tested package. Additionally, the number and diameter of holes in each package and the amount of damage sustained to the energy bar was determined. Hole diameter was measured by a stereomicroscope fitted with a calibrated ocular micrometer. The holes were also examined for fraying or scratches around the edges of each entry point to determine if the larvae created the hole, or if there was an undocumented defect in the package that allowed larvae to invade the package rather than penetrate it. In order to quantify and categorized the damage sustained to the packaged energy bar, a score of 0 to 4 was used. A score of 0 represented no visible damage to the energy bar. A score of 1 indicated that the bar had 1–25% of the total surface area covered with larval webbing, cast skins, frass, dead insects, and signs of feeding on the bar (damage). A score of 2 indicated that the damage ranged from 26–50% of the total surface area. A score of 3 represented 51–75% damage of the surface, and a score of 4 represented 76–100% damage of the surface area.

### 2.4. Experiment 2: Susceptibility of Packaging to Penetration by First, Third, and Fifth Instar P. interpunctella

Male and female moths collected from cultures were introduced into an inverted 0.95-L glass jar fitted with a mesh screen. Adult moths were allowed to mate and oviposit, and eggs collected (≤24 h) were added to 500 g of a poultry mash diet in a 0.95-L glass jar and held in a growth chamber at 28 °C and 65% r.h. to facilitate larval development. Eggs were used to represent first instars. An egg density of 50 eggs was chosen based on the results of experiment 1. The effect of larval density demonstrated little effect on the penetration ability of the first instars. Fifty eggs were counted under a stereomicroscope and added to individual 0.45-L glass jars, as described in experiment 1, with lids fitted with mesh screens and filter papers containing one of the three packaging types, Test A, B, or C. Egg hatch was determined as described in experiment 1. The mean ± SE (*n* = 3) egg hatch was 87.0 ± 1.7%.

Third and fifth instars were determined by measuring the head capsule width under a stereomicroscope [14]. Fifty third or 50 fifth instars, from culture jars, were added to separate 0.45-L glass jars containing one of the three packaging types—Test A, B, or C—and held at 28 °C and 65% r.h. Jars infested with eggs were examined at 21 days to count number of larvae and at 42 days to count the number of larvae, pupae, and adults found inside each package type. In the case of third instars, 21 days and 42 days observations included counts of larvae, pupae, and adults. Fifth instar observations were made after 21 days only, to count the number of larvae, pupae, and adults. A 42-d observation was not conducted, because after 42 days, *P. interpunctella* would have completed a life cycle, and penetrations of packaging material could not be distinguished between larval stages. After 21 days and 42 days, packages were assessed for diameter and the number of holes present, and the bars were rated for damage as described in experiment 1. Each package type, larval age, and exposure period combination was replicated five times. Three life stages of larvae were used to determine differences among larval ages in their respective penetration ability.

### 2.5. Experiment 3: Life Cycle Assessment of Packaging Penetration by P. interpunctella

Eggs of *P. interpunctella* were collected from cultures and introduced into glass jars containing the rearing diet, as described previously. In the 0.95-L glass jars, corrugated paper spools were added to the top of the diet to serve as pupation sites for wandering larvae [24]. Pupae were collected from the paper spools, and sexes were separated using the characteristics and illustrations described in the literature [25]. Two male and two female pupae were added to 0.45-L glass jars fitted with mesh screens containing one of the three packaging types—Test A, B or C—and held at 28 °C and 65% r.h. for 21 days and 42 days. After 21 days and 42 days, packages and bars were assessed as described previously. Each package type, and exposure period combination, was replicated eight times. *P. interpunctella* pupae were used as an alternative for male and female moths, in order to prevent handling damage to moths. This test would be analogous to *P. interpunctella* infestations in retail environments.

### 2.6. Data Analysis

For all of the experiments, a completely randomized design was used. The means and standard errors for the number of larvae, pupae, and adults that emerged and for bar damage, hole size, and number of holes were calculated and reported [26]. The number of larvae, pupae, or adults found inside each energy bar packages after 21 days and 42 days were transformed to log_10_ (x + 1) scale for further analysis. Data obtained on the number of holes in packages, hole diameter, and damage score were not transformed. Data collected from tests at the three first instar densities were subjected to two-way analysis of variance (ANOVA) at each observation time (21 days or 42 days) to determine the significant differences in each of the dependent variables measured, as influenced by first instar densities, the packaging type, and their interaction. Data collected from first, third, and fifth instars were analyzed similarly by observation time to determine the significant differences in each of the dependent variables measured among instars, the packaging type, and the interaction of these two main effects. Means for the life cycle assessment were subjected to a one-way ANOVA, and package type was the main factor.

## 3. Results

### 3.1. Experiment 1: Effect of Larval Density on the Penetration Ability of First Instar P. interpunctella

After 21 days and 42 days following the addition of *P. interpunctella* eggs, the percentage of packages penetrated varied by packaging type (Table 1). With the exception of the lowest egg density, *P. interpunctella* larvae penetrated more packages after 42 days exposure. The increase in package penetration between 21 days and 42 days can be associated with an increase in exposure time and instar age. The variation and lack of consistency in packaging penetration could be a result of *P. interpunctella* larval cannibalism, due to the lack direct access to a food source.

A two-way ANOVA of 21 days data show that the number of larvae found inside the packages was significantly different among egg densities (*F* = 4.03; df = 2, 36; *p* = 0.0263), but not among packaging types (*F* = 2.94; df = 2, 36; *p* = 0.0656). However, the egg density and package type interaction was significant (*F* = 6.85; df = 4, 36; *p* = 0.0003). Test B had the most larvae inside the package, 4.0 ± 3.1, when challenged with 50 eggs (Table 2). Test C was the only package penetrated at the 400-egg density level, which resulted in a higher larval count inside the package. The number of holes in the packages was not significant for egg density (*F* = 1.91; df = 2, 36; *p* = 0.1629), package type (*F* = 1.91; df = 2, 36; *p* = 0.1629), or their interaction (*F* = 2.59; df = 4, 36; *p* = 0.0529). The size of the holes produced in the packages was significant for egg density (*F* = 3.32; df = 2, 36; *p* = 0.0474), but was not significantly different for packaging type (df = 2, 36), or package type and egg density interaction (df = 4, 36) (*F*_range_ = 0.21–0.50; *p*_range_ = 0.7393–0.8135). The hole sizes ranged from 0.2–0.4 mm, which is within the range of head capsule sizes for first and second instars [14]. This indicates that the newly hatched larvae are capable of penetrating these packaging types. The damage score sustained by the energy bars was not significant for egg density (df = 2, 36) or packaging type (df = 2, 36) (*F*_range_ = 1.68–2.88; *p*_range_ = 0.0691–0.2007), but was significant for the package type and egg density interaction (*F* = 3.84; df = 4, 36; *p* = 0.0106). Test C packages subjected to 400 eggs sustained the most damage to the energy bar, which was a result of the higher amount of larvae present inside the package compared with the number of larvae found in the packages challenged with 50 eggs.

Statistical analysis of 42 days data showed that the number of larvae present inside the packages was not significant for egg density (df = 2, 36), package type (df = 2, 36), or their interaction (df = 4, 36) (*F*_range_ = 0.66–1.17; *p*_range_ = 0.3409–0.5215). However, the number of pupae found inside the packages varied significantly by egg density (F = 3.79; df = 2, 36; *p* = 0.0322), but was not significant for packaging type (*F* = 1.10; df = 2, 36; *p* = 0.3449). The egg density and packaging type interaction was significant (*F* = 2.74; df = 4, 36; *p* = 0.0437). There were no differences in the number of adults among egg densities (*F* = 2.13; df = 2, 36; *p* = 0.1340), but significant differences were observed among packaging types (*F* = 4.67; df = 2, 36; *p* = 0.0158). The interaction between egg density and package type was not significant (*F* = 1.30; df = 4, 36; *p* = 0.2889). Test C had the highest number of adults present inside packages, 4.4 ± 3.0 (Table 2). The number of holes found in the packages was not significant for egg density (df = 2, 36), packaging type (df = 2, 36), or their interaction (df = 4, 36) (*F*_range_ = 1.00–2.00; *p*_range_ = 0.1501–0.4203). The size of the holes in the packages was not significant among egg densities (*F* = 2.59; df = 2, 36; *p* = 0.0889), but it was significant among packaging types (*F* = 4.32; df = 2, 36; *p* = 0.0208), and Test C had the largest average hole size of 0.8 mm. The interaction between egg density and packaging type was not significant (*F* = 1.53; df = 4, 36; *p* = 0.2131). The damage score of the bars was not significant for any factor: egg density (df = 2, 36), packaging type (df = 2, 36), or their interaction (df = 4, 36) (*F*_range_ = 1.14–2.17; *p*_range_ = 0.1287–0.3531). Among the packaging types, Test C was the least resistant to penetration by *P. interpunctella* larvae after 21 days or 42 days, irrespective of egg density.

### 3.2. Experiment 2: Susceptibility of Packaging to Penetration by First, Third, and Fifth Instar P. interpunctella 

The third and fifth instars of *P. interpunctella* penetrated more packages than the first instars, regardless of the packaging type (Table 1). Additionally, the number of packages penetrated at 42 days either remained constant or showed an increase of up to 40% compared to 21-day penetrations. Among the packaging types, Test A had the least number of packages penetrated by the first and third instars after 21 days and 42 days of exposure. In tests conducted with fifth instars, Test B package had the fewest penetrated packages. The increase in the number of packages penetrated between 21 days and 42 days can be associated with the increase in the age of instars. As *P. interpunctella* increase in larval age, the mandibles of the larvae increase in strength, which may enable them to easily chew through packaging material.

A two-way ANOVA of 21 days data showed that the number of larvae found inside the packages was significant for variations in instar (*F* = 4.78; df = 2, 36; *p* = 0.0144), but was not statistically significant among package types (*F* = 2.25; df = 2, 36; *p* = 0.1196). The package type and instar interaction was not significant (*F* = 2.48; df = 2, 36; *p* = 0.0613). Test A had the least amount of larvae present inside the packages, and Test C had the most: 0 and 21.6 ± 9.3, respectively (Table 3). The number of pupae found inside the package was significantly different among instars (*F* = 17.99; df = 2, 36, *P* < 0.0001), package type (*F* = 6.23, df = 2, 36; *p* = 0.0047), and package type and instar interaction (*F* = 2.75; df = 2, 36, *p* = 0.0332). Similarly, the number of adults found inside the package was significant for instar (*F* = 9.50; df = 2, 36; *p* = 0.0005) and package type (*F* = 2.75; df = 2, 36; *p* = 0.0332). The package type and instar interaction was not significant (*F* = 2.20; df = 2, 36; *p* = 0.088). The increase in larval age (third and fifth instars) resulted in having a greater number of pupae and adults inside each package type. This was expected, because these instars are further along in their life cycle, while 21 days was not an adequate amount of time for the first instars to reach the pupal or adult stage. Additionally, the number of holes in the packages, the size of the holes, and the amount of damage to the energy bar increased with increasing larval age. Test C had the most holes per package, the greatest size of holes, and the highest damage score among the packaging types. The number of holes in the packages was not significant among the different stages of instars (*F* = 2.40; df = 2, 36; *p* = 0.1055). However, differences were significant among the packaging types (*F* = 9.37; df = 2, 36; *p* = 0.0005). The instar and packaging type interaction was also significant (*F* = 2.88; df = 2, 36; *p* = 0.0361). The hole size in the packages was significant among the different stages of instars (*F* = 4.76; df = 2, 36; *p* = 0.0147), but not among packaging types (*F* = 2.91; df = 2, 36; *p* = 0.0675). The interaction between instar age and packaging type was significant (*F* = 5.09; df = 2, 36; *p* = 0.0023). The hole sizes ranged from 0.3 mm to 1.3 mm. The wide range in hole sizes could be due to multiple larvae using the same entry point to gain access into the packaging material. Multiple larvae could chew at the same place in the package, thus causing the entry point to widen. The larger hole sizes were seen in packages challenged with third and fifth instars. The larger holes may be indicative of the size needed for adult moths to leave the inside of the package after emerging. The energy bar damage score was not significant among the different stages of instars (*F* = 2.18; df = 2, 36; *p* = 0.1283). However, packaging type significantly influenced the amount of damage sustained to the energy bars (*F* = 6.54; df = 2, 36; *p* = 0.0038). The instar and package type interaction was significant (*F* = 4.25; df = 2, 36; *p* = 0.0064).

Analysis of 42 days exposure results only include data for first and third instars, because after 42 days, fifth instars would have completed the development to adulthood, and therefore it would be difficult to determine which life stages found inside the packages were a result of the original larvae or from the next generation of larvae. Furthermore, the numbers of larvae that were present inside the test packages for first and third instars were recorded as present or absent, because after 42 days of testing, there were larvae present in various life stages of development from eggs laid by F_1_ adults. The first instars in Test A were the only package that did not have larvae present inside the packages (Table 3). Statistical analysis showed that the number of pupae found inside the packages was not significantly different among instars (df = 1, 24) package types (df = 2, 24), or their interaction (df = 2, 24) (*F*_range_ = 0.00–3.23; *p*_range_ = 0.0572–1.000). Similarly, the same trend was seen in the number of adults found inside the packages (*F*_range_ = 0.23–3.13; *p*_range_ = 0.0619–0.6373). The number of holes was not significant for instar (*F* = 2.70; df = 1, 24; *p* = 0.1132), but packaging type was statistically significant (*F* = 5.30; df = 2, 24; *p* = 0.0124). The interaction between instar and package type was not significant (*F* = 3.03; df = 2, 24; *p* = 0.0673). Test A was the most resistant to package penetration. The size of the holes in the packages varied, but the results were not significant among instars (df = 1, 24), package type (df = 2, 24), or their interaction (df = 2, 24) (*F*_range_ = 0.05–3.17; *p*_range_ = 0.0602–0.8290). The amount of damage sustained to the energy bars was not significant based on instars (*F* = 0.02; df = 1, 24; *p* = 0.8962). However, damage was significant for packaging type (*F* = 5.97; df = 2, 24; *p* = 0.0079), but the interaction between package type and instar was not significant (*F* = 1.58; df = 2, 24; *p* = 0.2261). In the first instar packages, the amount of damage sustained to the energy bar increased between 21 days and 42 days. 

### 3.3. Experiment 3: Life Cycle Assessment of Packaging Penetration by P. interpunctella

The number of packages that were penetrated either increased or remained constant from 21-d to 42-d exposure when subjected to male and female moth pairs (Table 1). Test A was the only package that was not penetrated after 21 days, but 75% of packages were penetrated by 42 days. Test C had the most packages penetrated at day 21—62.5%—but the percentage of packages that were penetrated remained constant after 42 days of exposure. The same trend was seen with Test B packages.

The number of larvae found inside the packages after 21 days was significantly different among packaging types (*F* = 5.33; df = 2, 42; *p* = 0.0087). Test C had the most holes in the packages, the largest hole size, and the greatest amount of damage to the energy bars (Table 4). The number of holes in the packages was not significant (*F* = 0.12; df = 2, 42; *p* = 0.8847). In addition, the size of the holes was not significant based on the packaging type (*F* = 0.75; df = 2, 42; *p* = 0.4807). The amount of damage that was sustained to the energy bars was also not significant based on the packaging type (*F* = 1.85; df = 2, 42; *p* = 0.1693). After 42 days, all of the samples had at least 25% of the packages penetrated. An analysis of the data showed that there was no statistical difference for the number of larvae (df = 2, 21), pupae (df = 2, 21), or adults (df = 2, 21) that were found inside the packages (*F*_range_ = 0.50–1.00; *p*_range_ = 0.3847–0.6149). Additionally, the number of holes in the packages (df = 2, 21), the size of the holes (df = 2, 21), and damage scores (df = 2, 21) were not statistically significant (*F*_range_ = 0.50–2.36; df = 2, 21; *p* = 0.1185–0.6136). However, the size of holes and the damage scores increased between 21 days and 42 days.

## 4. Discussion

Insect contamination during the post-processing and storage of food products is the origin of many consumer complaints to food manufacturers. The primary way of preventing package infestations is by employing insect-resistant packaging [27]. The objective of this research was to evaluate the susceptibility of three types of polypropylene-packaged energy bars to penetration and infestation by *P. interpunctella* larvae, and present methods to test packaging integrity. This study demonstrated that first, third, and fifth instars of *P. interpunctella* are all capable of penetrating undamaged multilayer polypropylene energy bar packages with combined total thicknesses ranging from 40.6–48.7 μm, after 21 days of exposure. Similarly, dry milk packages with no defects were still susceptible to infestations by *Attagenus unicolor* (Brahm), the black carpet beetle, and *Trogoderma glabrum* (Herbst)*,* the glabrous cabinet beetle [27]. On the contrary, *Sitophilus zeamais* Motschulsky, the maize weevil, were not able to penetrate 16 different pasta package types when no mechanical air vent microholes were present [28]. However, *S. zeamais* invaded polypropylene packages that had microholes ranging in size from 1.63 mm to 2.5 mm [28].

Our results document that although first instar *P. interpunctella* are typically invaders, they will penetrate packaging material when provided the opportunity. Our study also showed that older instars of *P. interpunctella* are capable of causing more damage to energy bars compared with first instars, presumably because the latter instars have a greater probability of penetrating polypropylene package due to their large and powerful mandibles compared with first instars. Previous research demonstrated that the ability of *P. interpunctella* larvae to penetrate packaging materials varied with age and packaging type. In general, all of the previous research has shown that older larvae penetrated more packaging material compared with younger larvae [19,20,22,29].

One study found that second and fifth instars were unable to penetrate 25.4 µm thick polypropylene films, but were able to penetrate 25.4 µm thick polyethylene films [19]. Another study found that first and fifth instars were unable to penetrate 28 µm polypropylene pouches, but could penetrate 25 μm polyvinyl chloride films when pouches were exposed to larvae for five days [20]. These two studies tested single-layer films that were roughly half of the total package thickness that was used in this study. A third study demonstrated that newly hatched larvae are unable to penetrate 20 µm polyethylene film even after 2–3 weeks of exposure [18]. Our study showed that penetration in Test B and C packaging occurred after 21 days of exposure, but the number of packages penetrated increased after 42 days of exposure. In the previous studies, the time that the larvae were exposed to packaged films was shorter compared to this study [18,20]. The additional time in this study indicates that larvae exposed to surfaces for an extended period of time may increase their ability to penetrate packages. Only using a five-day exposure to packages, third instars were not able to penetrate 20 μm casted polypropylene and oriented polypropylene packages [30]. The current study demonstrated that an increased exposure time—21 days compared with 42 days—increased the number of packages that were penetrated by first, third, and fifth instars. Again, the results of this study differ from previous studies, but further emphasize that exposure time is an important factor influencing the ability of *P. interpunctella* larval penetration, especially in the commercial environment, where the length of time that a package is on a store shelf or a warehouse could be upwards of several months.

Attention needs to be given towards the make-up of multilayer films such as the polymer blend, extrusion versus adhesion versus laminated processing, and metallized versus aluminum layer. The overall thickness of these packaging materials tested was thicker than those used in previous studies, but the individual layers were similar to those used in previous research. Despite the thicker material, *P. interpunctella* larvae consistently penetrated the energy bar packages, whereas in single-layer films, penetration was minimal. The results of this study demonstrated that increasing the film thickness decreased the ability of *P. interpunctella*’s ability to penetrate packages. Test C had the thinnest film—40.64 µm total thickness—and was consistently penetrated by all of the stages of *P. interpunctella* tested. In tests with first, third, and fifth instars, Test C had the highest larval, pupal, and adult counts found within the package. Additionally, Test C had the most holes per package, and the most damage to the energy bars after 21 days and 42 days exposures. Both Test A and B were consistently lower on all of the variables measured. Thus, thicker polypropylene films provide better protection against *P. interpunctella* penetration. However, the multilayer film used in this study is still not immune to penetration by larvae.

Packaged food products are susceptible to infestation all along the marketing and supply chain channels, especially if the packaging is permeable to food odors [31]. This study demonstrated that all of the stages of *P. interpunctella* are capable of penetrating energy bar packages when held without food. This experimental study most closely resembles the stored-product issues in the retail and warehouse environments compared with previous research, and provides a framework and methodology for testing packaging integrity. Further studies are warranted to determine the minimum thickness that can discourage *P. interpunctella* penetration on various substrates and among instars, as well as the volatility of food odors through the packaging material. This study provides the framework for testing new iterations of packaging against *P. interpunctella* penetration, and could be used as part of a prerequisite testing program for future packaging development.

## 5. Conclusions

*Plodia interpunctella* is a cosmopolitan stored product insect that is capable of invading and penetrating a variety of packaging materials. Our study demonstrated that multilayer polypropylene packaging is susceptible to penetration by *P. interpunctella* larvae by first, third, and fifth instars after 21 or 42 days. All packaging materials tested were susceptible to penetration, however the thickest material was most resilient. The methodology presented in our study provides the framework for future packaging integrity studies using *P. interpunctella* and other stored product insects.

## Figures and Tables

**Table 1:** Percentage of packages penetrated by *P. interpunctella* in each of the three experiments.

	Experiment 1 ^a^			Experiment 2 ^a^			Experiment 3 ^c^	
Trt	Egg Density	21 Days	42 Days	Instar	21 Days	42 Days ^b^	21 Days	42 Days
Test A	50	20%	0%	1	0%	20%	0%	75%
Test B		40%	0%		20%	60%	25%	25%
Test C		20%	0%		60%	80%	62.5%	62.5%
Test A	200	0%	0%	3	40%	80%		
Test B		0%	20%		60%	80%		
Test C		0%	40%		80%	80%		
Test A	400	0%	20%	5	80%	-		
Test B		0%	0%		0%	-		
Test C		60%	60%		100%	-		

^a^ A total of five packages was tested; ^b^ All of the adults from fifth instar larvae emerged with 21 days; ^c^ A total of eight packages was tested at each observation time.

**Table 2:** Number of larvae, pupae, and adults of *P. interpunctella* found inside the packages of energy bars after 21 days and 42 days of infestation, and the extent of damage to packages and energy bars.

Trt	Egg Density	Mean ± SE ^a^											
		Number of Larvae		Number of Pupae		Number of Adults		Number of Holes		Hole Size (mm)		Damage Score	
		21 Days	42 Days	21 Days	42 Days	21 Days	42 Days	21 Days	42 Days	21 Days	42 Days	21 Days	42 Days
Test A	50	2.4 ± 2.4	0.0 ± 0.0	--- ^b^	0.0 ± 0.0	--- ^b^	0.0 ± 0.0	0.2 ± 0.2	0.0 ± 0.0	0.3 ± 0.3	0.0 ± 0.0	0.4 ± 0.4	0.0 ± 0.0
Test B		4.0 ± 3.1	0.0 ± 0.0	---	0.0 ± 0.0	---	0.0 ± 0.0	0.4 ± 0.2	0.0 ± 0.0	0.4 ± 0.3	0.0 ± 0.0	0.6 ± 0.4	0.0 ± 0.0
Test C		0.2 ± 0.2	0.0 ± 0.0	---	0.0 ± 0.0	---	0.0 ± 0.0	0.2 ± 0.2	0.0 ± 0.0	0.3 ± 0.3	0.0 ± 0.0	0.2 ± 0.2	0.0 ± 0.0
Test A	200	0.0 ± 0.0	0.0 ± 0.0	---	0.0 ± 0.0	---	0.0 ± 0.0	0.0 ± 0.0	0.0 ± 0.0	0.0 ± 0.0	0.0 ± 0.0	0.0 ± 0.0	0.0 ± 0.0
Test B		0.0 ± 0.0	0.4 ± 0.4	---	0.8 ± 0.8	---	1.0 ± 1.0	0.0 ± 0.0	0.4 ± 0.4	0 .0± 0.0	0.2 ± 0.2	0.0 ± 0.0	0.8 ± 0.8
Test C		0.0 ± 0.0	0.0 ± 0.0	---	0.0 ± 0.0	---	4.4 ± 3.0	0.0 ± 0.0	0.6 ± 0.4	0.0 ± 0.0	0.6 ± 0.4	0.0 ± 0.0	1.2 ± 0.8
Test A	400	0.0 ± 0.0	3.2 ± 3.2	---	1.0 ± 1.0	---	0.0 ± 0.0	0.0 ± 0.0	0.2 ± 0.2	0.0 ± 0.0	0.2 ± 0.2	0.0 ± 0.0	0.8 ± 0.8
Test B		0.0 ± 0.0	0.0 ± 0.0	---	0.0 ± 0.0	---	0.0 ± 0.0	0.0 ± 0.0	0.0 ± 0.0	0.0 ± 0.0	0.0 ± 0.0	0.0 ± 0.0	0.0 ± 0.0
Test C		12.8 ± 4.6	0.0 ± 0.0	---	2.6 ± 1.1	---	2.4 ± 1.2	1.0 ± 0.5	0.6 ± 0.2	0.2 ± 0.1	0.8 ± 0.3	1.2 ± 0.4	1.8 ± 0.8

^a^ A total of five packages was exposed at each treatment and instars densities; ^b^ No pupae or adults were found at 21 days; only larvae were found at 21 days.

**Table 3:** Number of larvae, pupae, and adults of *P. interpunctella* found inside packages of energy bars after 21 days and 42 days of infestation with first, third, and fifth instars, and extent of damage to packages and energy bars.

Trt	Instar	Mean ± SE ^a^											
		Number of Larvae		Number of Pupae		Number of Adults		Number of Holes		Hole Size (mm)		Damage Score	
		21 Days	42 Days ^b^	21 Days	42 Days	21 Days	42 Days	21 Days	42 Days	21 Days	42 Days	21 Days	42 Days
Test A	1	0.0 ± 0.0	N	0.0 ± 0.0	0.0 ± 0.0	0.0 ± 0.0	0.0 ± 0.0	0.0 ± 0.0	0.2 ± 0.2	0.0 ± 0.0	0.2 ± 0.2	0.0 ± 0.0	0.2 ± 0.2
Test B		15.4 ± 7.1	Y	0.0 ± 0.0	0.2 ± 0.2	0.0 ± 0.0	3.6 ± 2.7	0.8 ± 0.2	0.6 ± 0.4	0.7 ± 0.4	0.6 ± 0.4	1.4 ± 0.6	1.4 ± 0.9
Test C		21.6 ± 9.3	Y	0.0 ± 0.0	0.4 ± 0.2	0.0 ± 0.0	8.0 ± 3.3	1.0 ± 0.6	0.8 ± 0.2	0.3 ± 0.2	1.2 ± 0.3	1.6 ± 0.7	2.6 ± 0.7
Test A	3	1.8 ± 1.4	Y	0.6 ± 0.4	0.0 ± 0.0	2.8 ± 1.8	2.6 ± 1.5	0.4 ± 0.2	0.6 ± 0.2	0.4 ± 0.3	0.8 ± 0.3	1.0 ± 0.6	1.2 ± 0.6
Test B		0.8 ± 0.4	Y	2.6 ± 1.3	0.0 ± 0.0	3.4 ± 2.1	0.6 ± 0.6	1.2 ± 0.4	0.2 ± 0.2	1.1 ± 0.3	0.3 ± 0.3	2.2 ± 0.6	0.2 ± 0.2
Test C		0.8 ± 0.8	Y	2.4 ± 0.7	0.8 ± 0.6	6.0 ± 2.3	3.4 ± 1.9	2.0 ± 0.6	2.8 ± 1.1	1.2 ± 0.3	1.0 ± 0.3	2.2 ± 0.6	2.6 ± 0.7
Test A	5	0.2 ± 0.2	--- ^c^	2.6 ± 0.9	---	4.6 ± 2.1	---	1.2 ± 0.4	---	1.0 ± 0.2	---	1.8 ± 0.6	---
Test B		0.0 ± 0.0	---	0.0 ± 0.0	---	0.0 ± 0.0	---	0.0 ± 0.0	---	0.0 ± 0.0	---	0.0 ± 0.0	---
Test C		1.6 ± 1.0	---	4.4 ± 0.6	---	7.4 ± 3.1	---	2.8 ± 0.7	---	1.3 ± 0.2	---	3.2 ± 0.2	---

^a^ A total of five packages was exposed at each treatment and instars combination; ^b^ N, no larvae were present; Y, larvae were present most probably from eggs laid by F_1_ adults; ^c^ All adults emerged within 21 days.

**Table 4:** Number of larvae, pupae, and adults of *P. interpunctella* found inside packages of energy bars in treatments with two mating pairs after 21 days and 42 days of infestation, and the extent of damage to packages and energy bars.

Trt	Mean ± SE ^a^											
	Number of Larvae		Number of Pupae		Number of Adults		Number of Holes		Hole Size (mm)		Damage Score	
	21 Days	42 Days	21 Days	42 Days	21 Days	42 Days	21 Days	42 Days	21 Days	42 Days	21 Days	42 Days
Test A	0.0 ± 0.0	2.1 ± 1.1	---^b^	0.6 ± 0.4	---^b^	0.0 ± 0.0	0.0 ± 0.0	0.9 ± 0.2	0.0 ± 0.0	0.7 ± 0.2	0.0 ± 0.0	0.9 ± 0.3
Test B	5.1 ± 4.3	1.4 ± 1.2	---	0.3 ± 0.3	---	0.0 ± 0.0	0.5 ± 0.4	0.3 ± 0.2	0.1 ± 0.1	0.4 ± 0.3	0.4 ± 0.2	0.5 ± 0.4
Test C	15.0 ± 6.7	2.1 ± 1.1	---	1.5 ± 1.0	---	0.5 ± 0.5	1.0 ± 0.4	0.8 ± 0.3	0.3 ± 0.1	1.0 ± 0.3	0.5 ± 0.2	0.9 ± 0.3

^a^ A total of eight packages was exposed at each observation time; ^b^ No pupae or adults were found at 21 days; only larvae were found at 21 days.
